# *Candida krusei* Lung Abscess With Black Necrotic Aspirate

**DOI:** 10.1002/rcr2.70460

**Published:** 2026-01-05

**Authors:** Shan Kai Ing, Pon Ying Lau, Nga Hung Ngu

**Affiliations:** ^1^ Respiratory Unit, Department of Medicine Sibu Hospital Sibu Sarawak Malaysia

**Keywords:** black aspirate, *Candida krusei*, lung abscess, opportunistic fungal infection

## Abstract

A profoundly immunocompromised man developed a large cavitary lung lesion producing black, viscous aspirate, with culture confirming *Candida krusei* . This rare manifestation underscores that atypically dark aspirates from lung cavities should raise suspicion for invasive fungal infection and prompt early antifungal therapy in patients with severe cellular immunodeficiency.

## Graphical abstract

Black, viscous necrotic aspirate from a cavitary lung lesion revealed invasive *Candida krusei* infection in a patient with advanced HIV. Dark or tar‐like aspirates should prompt evaluation for fungal disease in immunocompromised hosts.

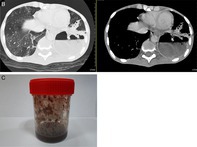

A 29‐year‐old man with newly diagnosed HIV infection (CD4 17 cells/μL) and chronic hepatitis B and C coinfection presented with progressive dyspnoea and dry cough shortly after discharge following treatment for Fournier gangrene and multidrug‐resistant bacterial pneumonia. Chest radiography demonstrated a large cavitary lesion with an air–fluid level in the left lower lobe (Figure 1A). Contrast‐enhanced CT of the thorax revealed diffuse bilateral ground‐glass opacities with tree‐in‐bud nodules and a thick‐walled 6 × 8 × 13 cm cavity surrounded by consolidation (Figure 1B). Ultrasound‐guided aspiration produced black, highly viscous necrotic material with sediment and surface film (Figure 1C). Direct microscopy revealed budding yeast cells, and fungal culture identified *Candida krusei* , an intrinsically fluconazole‐resistant species. Invasive *C. krusei* pulmonary infection is exceptionally rare and occurs mainly in individuals with profound cellular immunodeficiency, but non‐albicans *Candida* species are increasingly implicated in invasive candidiasis with rising antifungal resistance worldwide [1]. The unusual black discoloration of the aspirate likely reflected extensive tissue necrosis and fungal pigment deposition. In immunocompromised patients, cavitary lesions that yield dark or unusually viscous aspirate should prompt evaluation for invasive fungal infection and early targeted antifungal therapy [2].

**FIGURE 1:**
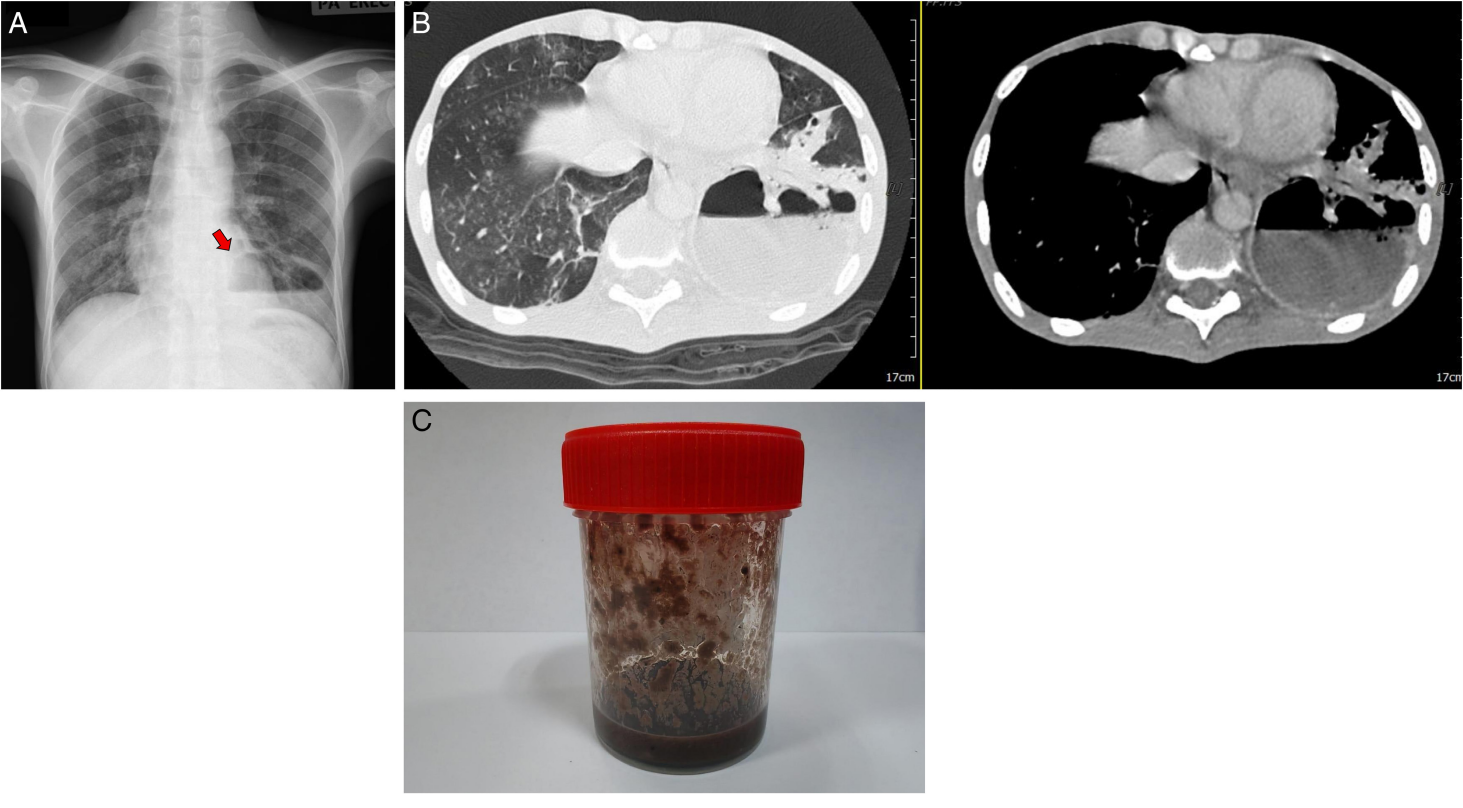
(A) Chest radiograph demonstrating a large cavity with an air–fluid level in the left lower lobe. (B) Contrast‐enhanced CT showing a thick‐walled 6 × 8 × 13 cm abscess cavity with surrounding consolidation and bilateral ground‐glass opacities. (C) Black, viscous necrotic aspirate obtained from the cavitary lesion; fungal culture yielded *Candida krusei* .

## Author Contributions

S.K.I. conceived the idea for case reporting and prepared the final manuscript with P.Y.L. and N.H.N. N.H.N. were the managing pulmonologists. All authors reviewed and approved the final version of the manuscript.

## Funding

The authors have nothing to report.

## Consent

The authors declare that written informed consent was obtained for the publication of this manuscript and accompanying images using the form provided by the Journal.

## Conflicts of Interest

The authors declare no conflicts of interest.

## Data Availability

Data sharing not applicable to this article as no datasets were generated or analysed during the current study.
